# A Fast Calibration Method for Phased Arrays by Using the Graph Coloring Theory

**DOI:** 10.3390/s18124315

**Published:** 2018-12-07

**Authors:** Lijie Yang, Ruirui Dang, Min Li, Kailong Zhao, Chunyi Song, Zhiwei Xu

**Affiliations:** Key Laboratory of Ocean Observation-Imaging Testbed of Zhejiang Province, Institute of Marine Electronic and Intelligent System, Ocean College, Zhejiang University, 316021 Zhoushan, China; yanglijie@zju.edu.cn (L.Y.); dangruirui@zju.edu.cn (R.D.); min_li@zju.edu.cn (M.L.); 21834139@zju.edu.cn (K.Z.)

**Keywords:** amplitude and phase errors, calibration, graph coloring theory, mutual coupling effect, planar phased array

## Abstract

Phased array radars are able to provide highly accurate airplane surveillance and tracking performance if they are properly calibrated. However, the ambient temperature variation and device aging could greatly deteriorate their performance. Currently, performing a calibration over a large-scale phased array with thousands of antennas is time-consuming. To facilitate the process, we propose a fast calibration method for phased arrays with omnidirectional radiation patterns based on the graph coloring theory. This method transforms the calibration problem into a coloring problem that aims at minimizing the number of used colors. By reusing the calibration time slots spatially, more than one omnidirectional antenna can perform calibration simultaneously. The simulation proves this method can prominently reduce total calibration time and recover the radiation pattern from amplitude and phase errors and noise. It is worth noting that the total calibration time consumed by the proposed method remains constant and is negligible compared with other calibration methods.

## 1. Introduction

To ensure a radiation pattern meeting the required performance, an active phased array must guarantee amplitude and phase matching among elements [1,2,3,4]. When these amplitude and phase errors are small and cannot be satisfied by the manufacturing process, the phased array antennas must be calibrated, especially for a digital beam-forming phased array with very sharp beams. Many calibration techniques have been proposed to obtain uniform amplitude and phase values for each antenna [3,5,6,7,8,9,10,11,12,13,14,15]. These methods can be divided into five types: Near-field calibration, auxiliary probe calibration, internal calibration, far-field calibration, and mutual coupling calibration. Near-field calibration uses a test probe scanning across the array surface to directly measure each antenna’s amplitude and phase errors. This method can obtain high-accuracy calibration results. However, it is very time-consuming since it requires a complex equipment to accurately control the probe’s movement. Near-field calibration is always performed in the factory and is not suitable for in-the-field calibration [6,9]. The auxiliary probe calibration method places auxiliary antenna probes around the array. These probes are used to couple radiant signal from each antenna. The received signal is compared to a stored reference obtained during factory test to obtain each antenna’s amplitude and phase errors. This method is much faster than the near-field calibration. However, the coupling between each probe and each antenna is different and always results in prominent calibration errors [10,11]. Internal calibration method places a built-in calibration circuitry beside each antenna to measure RF output power, receive gain and so on. These measured values are transmitted to a central calibration module where the amplitude and phase errors of each antenna are calculated. This method allows precise calibration by direct sampling of phases and amplitudes. A major disadvantage of this method is the requirement of significant additional hardware (high cost for large arrays) [3,12]. Far-field calibration method is a very popular method, which places a transmitter/receiver in the far field to transmit calibration signal to/receive signals from the array. This method can perform background calibration without interrupting radar’s normal operation. However, the calibration performance of this method is highly susceptible to environmental influence. Furthermore, it requires the radar keeping absolutely still during calibration. Sometimes the far-field transmitter/receiver is difficult to deploy. This method may not be optimum for airborne radars and ship-borne radars [2,13]. Mutual coupling calibration method uses the mutual coupling effect among antennas to measure the amplitude and phase differences by transmitting from one antenna and receiving from another. The mutual coupling among adjacent antennas should be identical and the array should be able to transmit with one antenna and receive with another simultaneously. Plenty of works have been down in this method. However, in these works, only one antenna transmits at a time, and this procedure is repeated until all antennas are measured [14,16].

Reference [16] combines the mutual coupling method and the rotating element electric field vector (REV) method [17] to achieve amplitude-only measurement for phased array calibration. By shifting the phase of one antenna from $0^{\circ }$ to $360^{\circ }$, the REV method measures the composite electronic field of the entire array and obtains the amplitude and phase for the antenna. However, the REV method’s total number of measurement is in proportion to the number of antennas and the phase shifting resolution. Assuming the bit number of the phase shifters is *p* for an array with *N* antennas, the total number of measurements using the REV method equals to $2^{p}N$, which is the measuring number of each antenna multiplied by the total antenna number [18]. If we divide the total calibration time into non-overlapping equal-length time slots and each time slot is allocated to a dedicated antenna, the number of measurements can also be expressed as the measuring number of each antenna multiplied by the number of time slots. This method consumes a large number of measurements to calibrate phased arrays with a large number of antennas, hence drastically reducing the effective radar working time. Some advanced algorithms have been proposed to reduce the number of measurements, such as the extended REV method [19] and the REV-H method [20]. The extended REV method measures signals of multiple antennas simultaneously. The phases of these antennas are successively shifted and then the relative amplitude and phase errors are obtained by expanding the measured power variation through Fourier transformation. However, the total number of measurements of the extended REV method is at least 7.5N+1. The REV-H method divides antennas into different groups according to the normalized Hadamard matrix. The phases of all the elements in the same group are rotated at the same time, and the composite electric field vector of this group can be obtained by the simplified REV method, through which we can derive the relative electric fields of all elements. This method requires 30N measurements. All these methods mentioned above aim to reduce the measuring number of every antenna. However, the total number of measurements is still proportional to the antenna number, which is still unacceptable for a large array.

In this paper, we propose a fast calibration method based on graph coloring theory. Other than the previously mentioned methods, which try to minimize the measurement number of each antenna, the proposed method focuses on sharing calibration time slots to calibrate multiple antennas simultaneously. Though limited literature has been published in the area of calibration time allocation for phased arrays, plenty of scheduling/resource allocation strategies in other areas can be found [21,22,23,24,25]. Reference [21] converts the intersensor interference avoidance problem into the scheduling problem of multiple access in a shared channel. It adopts the graph coloring theory to transform this scheduling problem into a coloring problem aiming at minimizing the number of used colors. Reference [22] proposes a pilot allocation scheme based on the graph coloring theory to mitigate pilot contamination for multi-cell massive MIMO systems. The reference firstly constructs an interference graph to describe the potential inter-cell interference relationship (ICI) among users. Then the graph coloring-based scheme is used to eliminate ICI in the interference graph. Reference [23] models a wireless sensor network (WSN) as an Interference-Communication (IC) graph and the receiver nodes or transmission links are assigned with different colors. Thus, it transforms the receiver-based and link-based interference-free channel allocation problems into graph coloring problems and proposes a fair channel allocation protocol that minimizes the maximum interference experienced by any transmission. The issue of phase array calibration is similar to the TDMA scheduling problem in three aspects. First, the goal of array calibration is to achieve the minimum calibration time under the condition that every antenna receives a calibration signal with minimal interference. The goal of a TDMA system is to achieve a minimal number of channels shared by nodes under the condition that every node can swap information without collision. Second, the calibration time slot available for each phased array antenna is analogous to the shared channel accessible for each node in the TDMA system. Third, multiple antennas occupying the same calibration time slot suffer from mutual interferences. Analogically, in the TDMA system, every node sharing the same channel will collide with each other. Graph colouring theory has already been widely used in the TDMA domain to solve this collision problem. Though no research concerning the use of graph theory in the phased array calibration is found, due to the similarity between phased array calibration and TDMA channel allocation, the graph colouring theory is theoretically applicable for the phased array calibration problem.

Compared to the REV method, the extended REV method, and the REV-H method, our proposed method achieves the minimum number of measurements, especially for large-scale phased arrays with omnidirectional radiation pattern. Extensive simulations demonstrate that the proposed method requires at most 8, 9 and 16 calibration time slots for a hexagonal-tiling planar array, square-tilting planar array, and triangular-tiling planar array, respectively.

The paper is organized as follows. In Section 2 and Section 3, the theory of the antenna scheduling method for phased array calibration is proposed, which adopts the graph coloring theory to transform the calibration scheduling problem into a coloring problem to minimize the number of colors. In Section 4, numeric simulations are provided to validate performance. Finally, Section 5 concludes the paper.

## 2. Problem

Phased array radars, especially digital beam forming based phased array radars, have demonstrated excellent performance. However, excessive amplitude and phase errors could deteriorate its performance. To minimize these errors, radar needs to be calibrated. There are several types of calibration. Factory calibration uses near-field scanning to identify errors, which is time-consuming and can only calibrate static errors initially. On-field calibration is often mandated after deployment to calibrate varied errors due to environment and aging. Our proposed calibration method can be applied to both situations. First, we leverage mutual coupling among omnidirectional antennas to perform local calibration based on the REV method. Second, we expand local calibration to as many antennas as possible with negligible interference, which can drastically reduce the overall calibration time.

**Figure 1:**
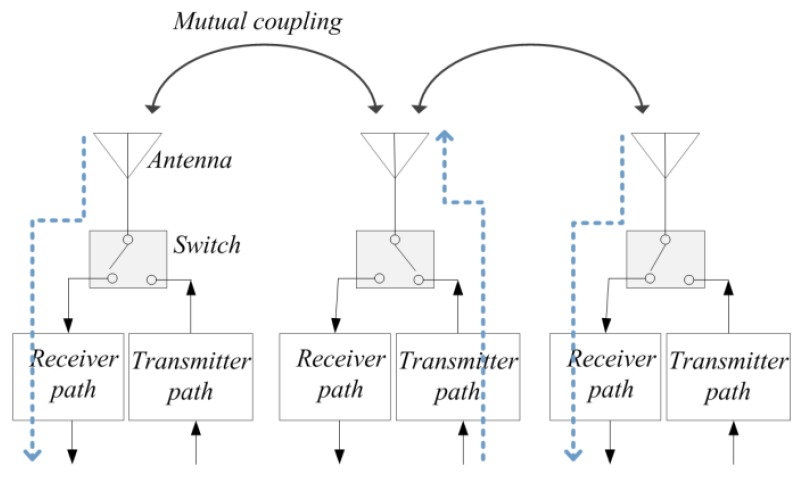
The antenna structure of a typical phased array. The receiver and transmitter share an identical antenna. The switch controls the transform between receiving mode and transmitting mode. A mutual coupling exists between adjacent antennas.

This paper targets to calibrate a large-scale planar phased array with omnidirectional antennas distributed regularly, which is demonstrated in Figure 1. The receiving path and transmitting path share an identical antenna. The switch controls the transition between transmitting and receiving modes. Below gives some definition for detailed discussion.

One-hop neighbor antenna: An antenna *j* is a one-hop neighbor of antenna *i* if the distance between antenna *i* and *j* is the shortest; Antenna *j* is also called the adjacent antenna of antenna *i*;

Two-hops neighbor antenna: An antenna *j* is a two-hop neighbor of antenna *i* if antenna *j* is the one-hop neighbor of antenna *i*’s one-hop neighbor.

*n*-hops neighbor antenna: An antenna *j* is an *n*-hops neighbor of antenna *i* if antenna *j* is the one-hop neighbor of antenna *i*’s (*n-1*)-hops neighbor.

**Figure 2:**
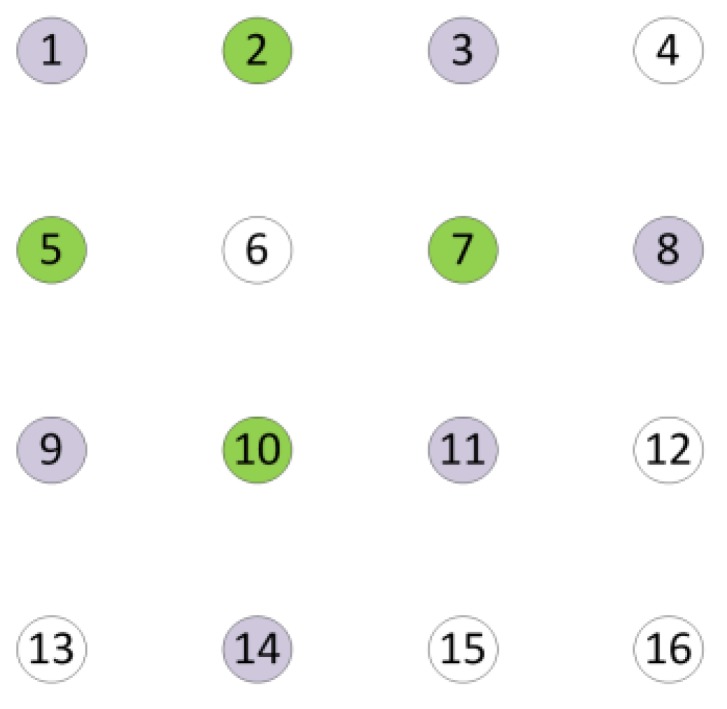
A uniform planar array with 16 antennas. The green circles represent one-hop neighbor antennas of antenna 6, and the grey circles represent two-hops neighbor antennas of antenna 6.

We take a regular planar array in Figure 2 as an example. Omnidirectional Antennas are represented by circles. The one-hop neighbor antennas of antenna 6 are antenna 2, 5, 7, and 10, and the two-hops neighbor antennas of antenna 6 are antenna 1, 3, 8, 9, 11, and 14.

### 2.1. Mutual Coupling

**Figure 3:**
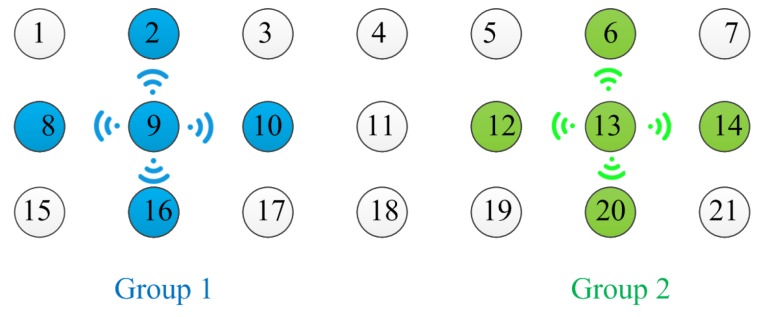
Demonstration of two groups of antennas performing local calibration simultaneously. These two groups are colored with blue and green. The central antennas (antenna 9 and 13) need to keep *m*-hops away from each other to nullify mutual interference.

Given omnidirectional antennas in a regular array with identical spacing between adjacent antennas, every antenna couples identical power to all its *n*-hops neighbor antennas and presents same mutual coupling coefficients. The coupled power decays with increasing distance. Therefore, any two groups of antennas must keep enough distance to reduce mutual interference when performing local calibration simultaneously, as shown in Figure 3, where antennas in group 1 receive interferences from antennas in group 2. In general, we can suppose that the mutual interference between two groups can be ignored when they are separated more than *m*-hops away from each other. The value of *m* is determined by the mutual coupling effect between antennas as well as the signal-to-interference ratio (SIR) required by each group to perform local calibration.

### 2.2. Local Calibration

Take Figure 3 as a simple explanation of the local calibration. First, the radar calibration controller turns an antenna, e.g., antenna 9, into transmission mode and radiating a calibration signal. The controller also turns all adjacent antennas (e.g., antenna 2, 8, 10, and 16) into receiving mode and receiving the radiant calibration signal. By using the mutual coupling-based REV method, the amplitude and phase errors among antenna 2, 8, 10, and 16 can be measured. The REV method measures amplitude of the composte signal of antennas in one group when phase of each antenna is shifted from 0${}^{\circ }$ to 360${}^{\circ }$. The measured amplitude is sinusoidal about the phase shift. The ratio of maximum amplitude value and minimum amplitude value is used to calculate the amplitude error of each antenna. The phase shifter corresponding to the maximum amplitude is used to calculate the phase error of each antenna. Detailed description of the REV method can be found in [15,16].

However, the interference from the other nearby transmitting antennas could deteriorate the mutual coupling-based calibration. For instance, when antenna 9 and its adjacent antennas are performing mutual coupling-based REV method, antenna 11 happens to be in transmitting mode. Antenna 10 receives signals from both antenna 9 and 11. The signal from antenna 11 is interference, which deteriorates the received calibration signal from antenna 9. Hence the conventional mutual coupling-based calibration method activates only one single transmitting antenna at a time, and all the other antennas switch to receiving mode, which is the root cause for the enormous calibration time. In order to cut calibration time, we propose an anti-interference calibration method by allocating antennas with significant mutual interference into other calibration time slots. Consequently, we can disregard the interferences from other nearby transmitting antennas, and receive the desired calibration signal with enough SIR. Consequently, we achieve flexibility to arrange many calibrations concurrently and reduce the total calibration time.

### 2.3. Calibration Time Slot Scheduling vs. Graph Coloring

A phased array contains thousands of antennas. Antenna failures may occur sporadically after a long-term operation, and lead to topology variation. In addition, antennas may also suffer from performance degradation. This must be considered in the calibration scheme.

Taking antennas as dots and the mutual couplings between one-hop neighbor antennas as lines, the calibration time slots scheduling issue of a phased array is transformed into a coloring problem. The objective is to minimize the total calibration time, which is equivalent to minimize the number of used colors for a map.

**Figure 4:**
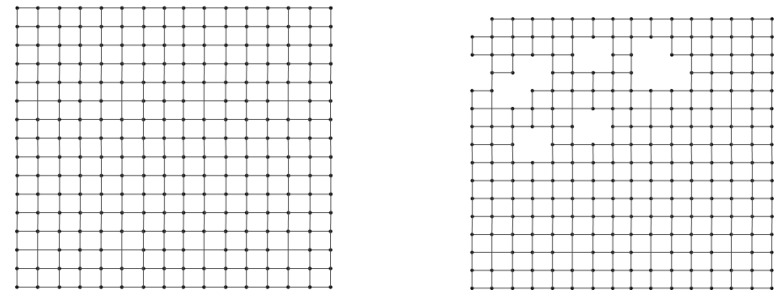
Demonstration of the topology changing for a square-tilting planar phased array with some antennas break down.

An example of a square phased array is shown in Figure 4a. When there are defect antennas, it is shown in Figure 4b, in which the defect antenna loses mutual couplings with its adjacent antennas. Hence there are no connection lines. Similar to the TDMA scheme [23,25,26], we divide the total calibration time into non-overlapping equal periodic time slots. These time slots are assigned to different antennas for local amplitude and phase calibration simultaneously. Based upon this, an interference-negligible antenna calibration scheduling scheme can be obtained by arranging as many antenna calibrations in one time slot as possible to achieve efficiency. This is similar to color a graph with minimum colors.

The graph coloring scheme has already been applied for channel assignment problems in the time, frequency and code domains [22,27,28]. Here, we use it to optimize phased array calibration schemes. Given a simple graph, *G* = (*V*, *E*), where *V* is the set of vertices (i.e., antennas) and *E* is the set of edges (i.e., mutual couplings between adjacent antennas). The one-hop neighbor vertices of each vertex *v* are defined as

$$N\left(v\right)=\left\{u:(u,v)\in E\}\right.$$

To arrange the calibration time slots of antennas is equivalent to color each vertex, which is essential to determine a color assignment strategy for *G*, that is f:V(G)→F, where *f* is the assignment function and *F* is a set of colors. The values in *F* are positive integers representing different colors so that any two vertices interfering with each other are given different colors. To improve the calibration efficiency is to minimize the number of colors used so as to achieve minimum time slots for calibration.

Since each antenna in Figure 1 is constituted of both transmitter and receiver, the calibration procedure for a single antenna can be divided into two parts, i.e., receiver path calibration and transmitter path calibration.

**Receiver path calibration**

Given any one vertex *v* in *V*, we turn *v* into transmitting mode and its one-hop neighbor vertices N(v) into receiving mode. Because of the existing of *E*, every vertex in N(v) can receive the calibration signal transmitted by *v*. Therefore, the local calibration techniques can be performed to obtain the amplitude and phase mismatches among vertices in N(v). There is one constraint that *v*’s neighbor vertices within *m*-hops are prohibited from transmission to minimize interference. Amplitude and phase errors of other vertices can be measured similarly.

**Transmitter path calibration**

Similarly, for the transmitter path calibration, the vertex *v* is turned into receiving mode, and antennas in N(v) are in transmitting mode so that the signal received by *v* is a composition of signals transmitted by N(v). Afterward, the local calibration can be applied to solve the amplitude and phase mismatches among antennas in N(v). Similar to the analysis in the receiver path calibration, here *v*’s neighbors within *m*-hops are prohibited from being in receiving mode.

Thus, the problem considered in this paper is to identify the optimal coloring solution for the following problem:

(1) $$\left\{\begin{array}{ll} \mathrm{Destination}:\;min|F| & \\ \mathrm{Condition}:\;f(u)\neq f(v), & \mathrm{when}\;u\;\mathrm{is}\;v\prime s\;\mathrm{neighbor}\;\mathrm{antenna}\;\mathrm{within}\;m-\mathrm{hops}\\ & \mathrm{and}\;u,v\in V. \end{array}\right.$$

According to [29], finding such an optimal solution for this problem is NP-complete. Therefore, it is critical to identify an effective algorithm.

## 3. Graph Coloring Theory-Based Array Calibration

In this section, we propose a fast array calibration method based on graph coloring theory, which does not require array’s global topology information. This scheme can also handle dynamic topology variation due to antenna failure or array upscaling. Therefore, the array equipped with the proposed algorithm can ensure large-scale phased array performance under harsh environments, like outer space, desert, and ocean surfaces. To be specific, every vertex *v* in *V* obtains the connection information of its neighbors within *m*-hops in phase one of the calibration. In phase two, they exchange time slots allocation information among neighbor vertices within *m*-hops to facilitate calibration. A detailed explanation is as follows.

**Phase one**

In this phase, we build up a neighbor map for every vertex to record its neighbors within *m*-hops.

**Figure 5:**
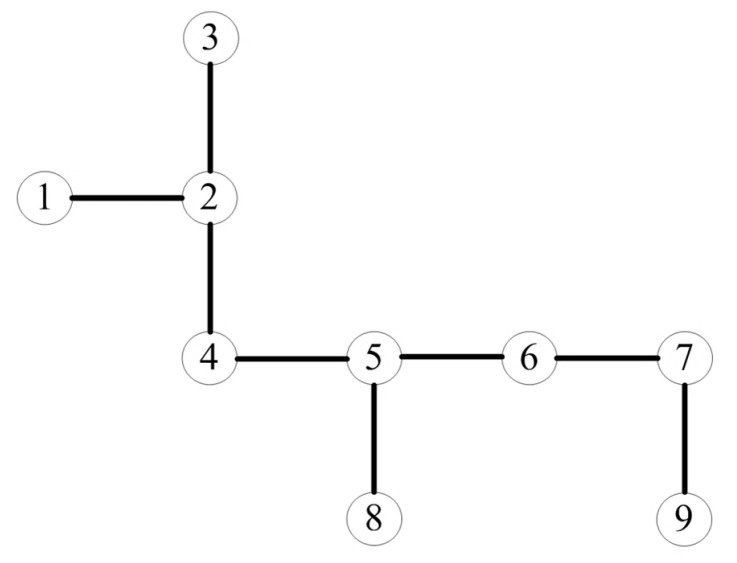
A topology example of 9 antennas (vertices). The circles represent antennas and lines represent mutual couplings between one-hop neighbors.

Each vertex does not have knowledge of its neighbors within *m*-hops initially, and the corresponding neighbor map is vacant for every vertex. Take the topology of the nine vertices array in Figure 5 as an example. For convenience and illustrative purposes, we assume that *m* is 3 in this example. Situations with different *m* can be deduced by analogy. The procedure to build a neighbor map is expressed as follows:

1. Label antennas (vertices) from 1 to 9;
2. Turn antenna 1 into transmitting mode and others into receiving mode; and
3. The antenna 1 broadcasts its identity information. Due to the existence of mutual coupling, all antennas receive this signal with different power level. The signal received by antenna 1’s one-hop neighbor antenna (i.e., antenna 2) is much higher than those from other antennas’. We then set up a signal detection threshold to ensure that the received signals from one-hop neighbor antennas exceed the threshold and are identified, while the received signals from other antennas are below the threshold and disregarded. By doing this, an antenna can only propagate its identity to one-hop neighbor antennas;
4. When antenna 2 receives this signal, antenna 2 records antenna 1 into its neighbor map as its one-hop neighbor. However, antenna 2 does not know its two-hop neighbors and three-hop neighbors;
5. Then antenna 2 turns into transmitting mode and broadcasts its identity while the others turn into receiving mode. This signal is received by antenna 1, 3, and 4, so that antenna 1, 3, and 4 add antenna 2 into their neighbor maps accordingly;
6. After all antennas have broadcasted their identities to their one-hop neighbors, all antennas have built up their neighbor maps with knowledge of their one-hop neighbors, as shown in Table 1.

Subsequently, we kick off the second round broadcasting to obtain knowledge of their two-hop neighbors. Similarly, each antenna broadcasts its identity together with the identities of their one-hop neighbors to enable the acquisition of antennas’ two-hop neighbors, as shown in Table 2. For instance, antenna 2 broadcasts a radio-frequency signal to the air containing its own identity as well as its one-hop neighbors’, i.e., antenna 1, 3, and 4. Antenna 1 receives this signal and knows that antenna 3 and 4 are antenna 2’s one-hop neighbors and hence antenna 1’s two-hops neighbors.

In the third round broadcasting, every antenna broadcasts its identity with the identities of their neighbors within two-hops. Therefore, every antenna obtains the information of its neighbors within three-hops, as shown in Table 3.

For situations with a different *m*, the aforementioned broadcasting procedure is repeated *m* times, until each vertex obtains the information of its neighbors within *m*-hops.

**Phase two**

In phase two, we use an allocation matrix to derive the number of time slots that each calibration needs, where the matrix’s row represents each vertex and the matrix’s column represents the allocated calibration time slots (colors). The maximum number of allocated calibration time slots equals the number of vertices when every vertex occupies a time slot. Each grid in the allocation matrix can have three values: 1, 0, and blank, which is defined as follows:

**1:** The vertex reserves the time slot for calibration;

**Blank:** The vertex can use this time slot for calibration; and

**0:** The vertex cannot use this time slot because it is reserved for the vertex’s neighbors within three-hops.

Obviously, the maximum number of calibration time slots is 9 for our example. This scheme has been adopted by the methods mentioned in [17,19,20] and is inefficient.

**Figure 6:**
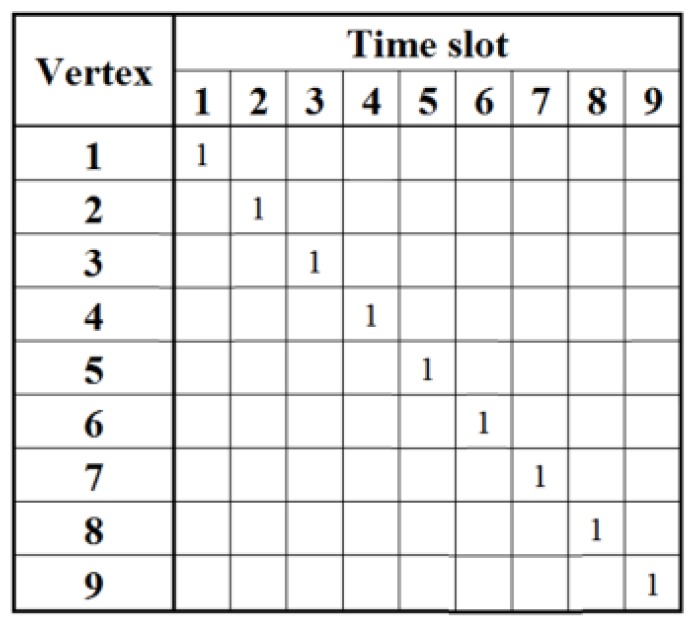
The initial state of the allocation matrix.

**Figure 7:**
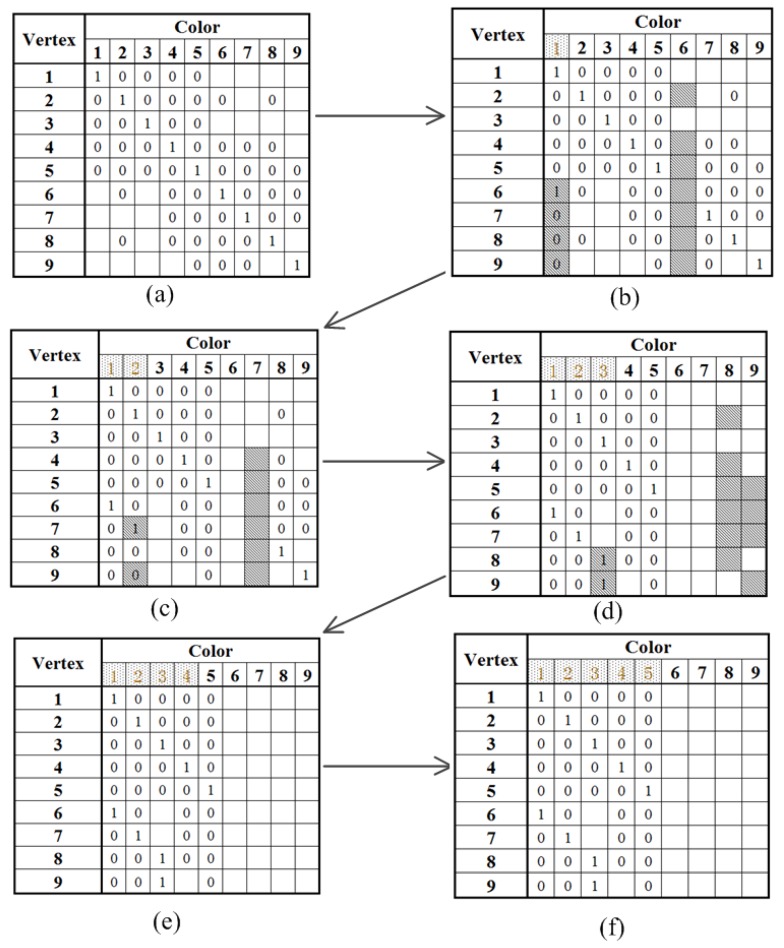
The step-by-step evolution of the allocation matrix. (**a**) depicts the initial allocation matrix after building up the neighbor map for every antenna. (**b**) shows vertices 1 and 6 are assigned with time slot 1. Time slot 6 is released. Time slot 1 is no longer available for vertices 7, 8, and 9. (**c**–**f**) demonstrate the assignment of time slots 2 to 5 among these vertices.

The allocation matrix starts with the initial state that the *i*-th time slot is reserved for the *i*-th vertex, as shown in Figure 6. Given the knowledge of its neighbors from phase one, the *i*-th vertex is able to infer the prohibited time slots that must be reserved for its neighbors within *m*-hops (*m* = 3 in this example). For instance, vertex 2 can deduce that time slots 1, 3, 4, 5, 6, and 8 are reserved for its neighbors within three-hops. It can also deduce that the remaining time slots are available to use. Moreover, vertex 2 also has partial knowledge of vertices 1, 3, 4, 5, 6, and 8’s neighbors. In particular, vertex 2 knows that time slots 1 and 4 are unavailable for vertex 3 since vertices 1 and 4 are vertex 2’s one-hop neighbors and vertex 3’s two-hops neighbors. However, vertex 2 does not know whether time slots 7 and 9 are available for vertex 3, because vertex 2 does not know whether vertices 7 and 9 are in vertex 3’s neighbor map or not. The deduced allocation matrix of every vertex after building up the neighbor map is shown in Figure 7a. This suggests that the total time slots are proportional to the number of antennas without any calibration time slot scheduling algorithm, and the calibration time is long.

The next step is to determine the time slot allocation strategy with negligible interference. For example, it is intuitive that vertices 1 and 6 can execute calibration concurrently in time slot 1, or vertices 1 and 7, vertices 1 and 8, vertices 1 and 9. Since there are many options, we use the following steps to identify the suitable combination.

First, every vertex broadcasts its own row information shown in Figure 7a. Hence, every vertex obtains the time slots allocation information of its own and its one-hop neighbors’. Second, every vertex broadcasts its own row information and its one-hop neighbors’. At last, every vertex broadcasts its own row information and its neighbors’ within two-hops. As a result, every vertex can acquire time slot allocation information of its neighbors within three-hops. For instance, vertex 8 knows that time slot 1 is unavailable for vertices 2, 4 and 5 while it is available for vertices 6, and 7. However, vertex 8 does not know whether time slot 1 is available for vertex 9 since vertex 9 is not in its neighbor map. When vertices 6, 7, 8, and 9 execute calibration in time slot 1, they may experience interference. To resolve this issue, an extra mechanism has to be applied to re-allocate time slot 1. We simply assign each vertex with a priority according to its vertex number. For example, vertex 6 has the priority over vertices 7, 8, and 9 to use time slot 1, and vertices 7, 8, and 9 clearly know this. When vertex 6 occupies time slot 1, time slot 6 is released and becomes available to other vertices. Vertex 6 broadcasts its new time slot occupation status to its neighbors within three-hops. As a result, vertex 2, 4, 5, 7, 8, and 9 can infer that time slot 1 is unavailable and time slot 6 becomes available. All these updates are illustrated in Figure 7b, where the time slot updates are highlighted in grey. Figure 7c to f show the assignments of time slots 2, 3, 4, and 5. It is worth noting that only five time slots are used to perform array calibration by using the proposed color assignment strategy.

**Figure 8:**
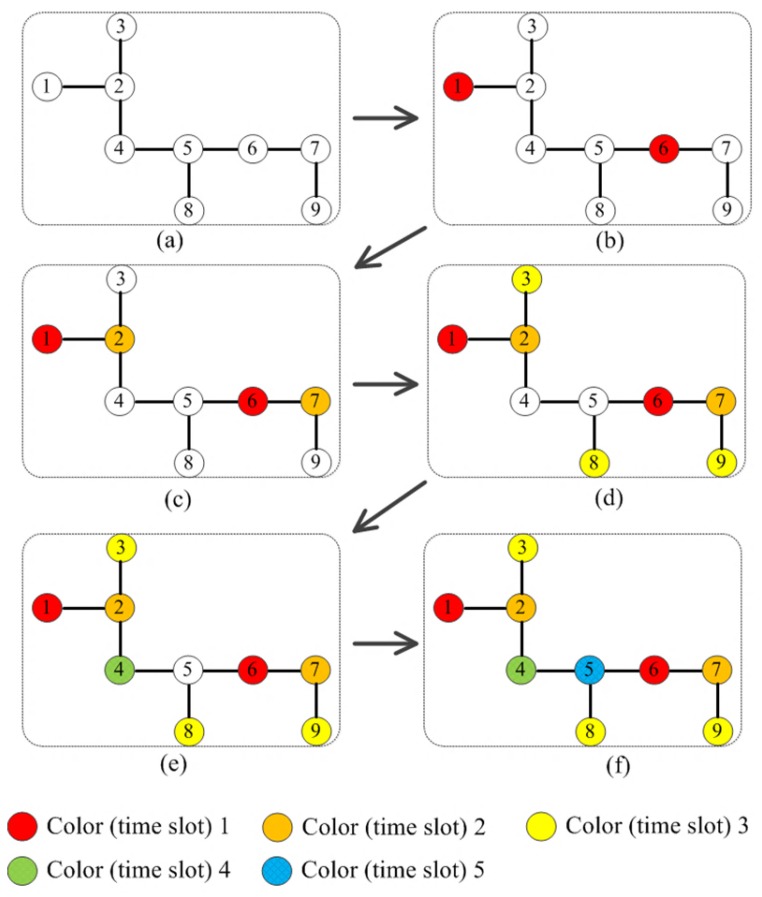
(**a**) The topology of 9 antennas without any color assignment. (**b**–**f**) depict the assignment of colors among antennas.

Figure 8 presents the color (time slot) assignment in the antenna topology to further clarify the proposed scheme. It is apparent that no any neighbors within three-hops are assigned with the same color (time slot).

## 4. Simulation

**Figure 9:**
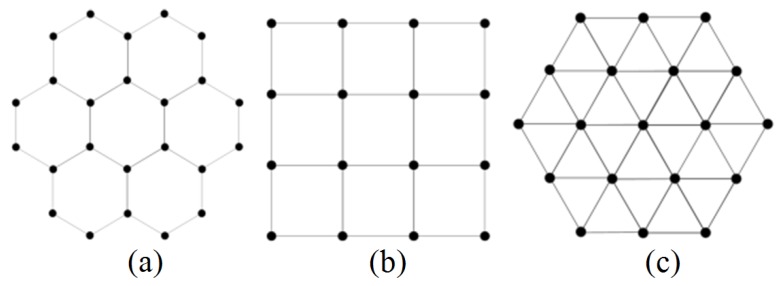
Three antenna topologies for planar arrays. (**a**) Hexagonal tiling. (**b**) Square tiling. (**c**) Triangular tiling.

We implement the proposed color assignment based calibration on several typical antenna topologies belonging to uniform phased array. Arrays with antennas scattered randomly are not covered in this paper. As shown in Figure 9, we discuss three antenna array topologies, i.e., hexagonal tiling, square tilting, and triangular tiling, according to the number of one-hop neighbors.

### 4.1. The Mutual Coupling vs. Antenna Separation

First, we evaluate mutual coupling between two circular patch antennas for different separations (*S*) in HFSS 15, which validates the assumption in Section 2.1 that the interferences are negligible compared to the calibration signal, given that, it meets the condition of the optimal coloring problem shown in Equation (1).

**Figure 10:**
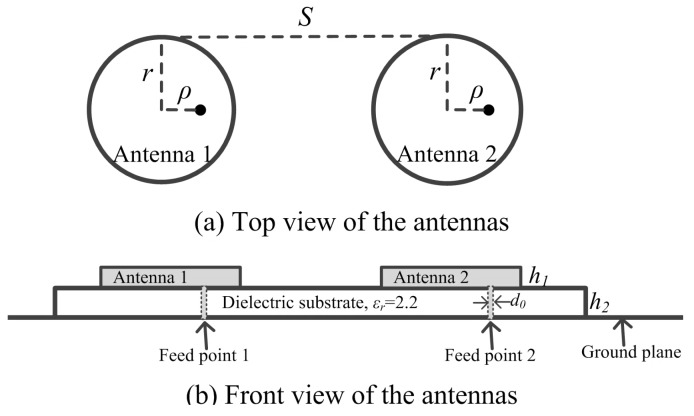
The dimension of antennas used to evaluate the mutual coupling effect, where *r* = 51.75 mm, ρ = 21.11 mm, $h_{1}$ = 0.1 mm, $h_{2}$ = 1.575 mm, $d_{0}$ = 0.65 mm.

Figure 10 shows the dimension of circular patch antennas in the first simulation. Various types of patch antennas with omnidirectional radiation patterns are available [30,31,32,33,34]. We chose a circular patch antenna [30] as the implementation architecture for validation.

**Figure 11:**
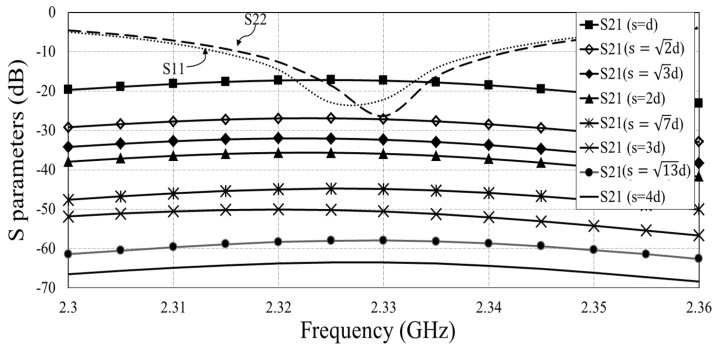
Simulated return loss (S11, S22) and mutual couplings (S21) versus frequency with different separation, *S*.

Figure 11 shows the simulated return losses (S11 and S22) and mutual couplings (S21) with different *S*. The resonant frequency is 2.33 GHz. The numerical mutual coupling results at 2.33 GHz are listed in Table 4. The mutual coupling decays about 18.56 dB when the separation *S* is increased from d to 2d (*d* = 105 mm). When S≥2d, the mutual coupling decays about 13 dB/*d*. The simulation results indicate that the received signal strength from a one-hop transmission antenna (S=d) is 46.16 dB stronger than that from a four-hop transmission antenna (S=4d). To achieve a better than 10 dB SIR for an acceptable accuracy of calibrated phase and amplitude mismatch, we mandate that two calibration groups are four-hops or more hops away from each other, as mentioned in Section 2.1. This essentially ensures the received calibration signal SIR, and we will discuss this in the following paragraph. It is worth noting that the mutual coupling between antennas depends on many factors, including antenna geometry, the separation between adjacent antennas, the substrate used, and so on. Arrays with stronger mutual coupling effect require larger *m* to obtain the desired SIR for each group.

### 4.2. Received Calibration Signal’s SIR

By applying the proposed color assignment method, multiple antennas are able to perform calibration simultaneously. Hence, every receiving-mode antenna receives calibration signals from its one-hop neighbor at transmitting-mode as the desired calibration signal, and from other multiple-hop neighbors at transmitting-mode as interferences. In the second simulation, we evaluate the received calibration signal’s SIR.

There are two extreme cases during calibration. One is when an antenna locates at the center of the array whose neighbors are symmetrical around it with the strongest interferences, and the other one is when an antenna located at the corner of the array whose neighbors are asymmetrical with longer distances and the least interferences. The other cases have asymmetrical surrounding antennas whose distances are between the above two cases. Here we define the distance between adjacent antennas as *d*. To simplify the analysis, we only need to evaluate the performance of these two extreme cases.

**Figure 12:**
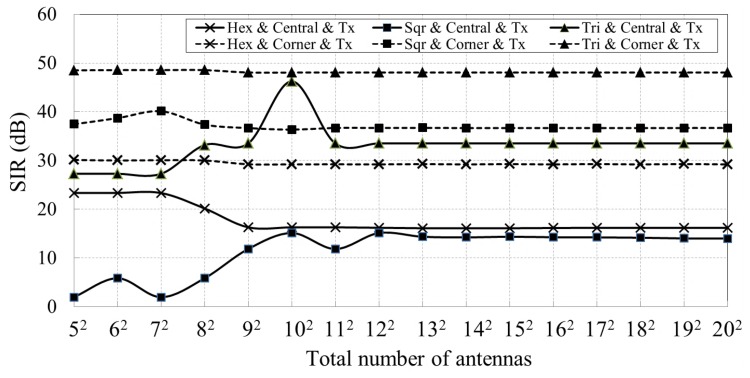
Received calibration signal’s SIR versus total number of antennas, where the solid lines show the calibration signal’s SIR when the central antenna is configured as a calibration signal transmitter. The dotted lines show the calibration signal’s SIR when the antenna at the top left corner of the array is the calibration signal transmitter.

First, we evaluate the case where the antenna located at the center of the array being activated as a transmitter to calibrate its one-hop neighbors. This corresponds to the receiver path calibration as described in Section 2.3. The calibration signal and interferences are generated at the feeding points of each transmitter antenna with an identical frequency and phase. After propagating through different distances, all interferences are superimposed at a one-hop neighbor antenna of the central antenna, which is in receiving mode and the received calibration signal’s SIR can be calculated. We implement this simulation over arrays with different topologies and different number of antennas, whose simulation results are represented by three solid lines in Figure 12. This is the worst condition because the antennas under calibration receive the strongest interference. Second, we evaluate the case where the antenna located at the corner (e.g., top left) of the array serves as the calibration signal transmitter and its one-hop neighbors are the antennas under calibration. We calculate the calibration signal’s SIR received by these antennas, whose results are shown as the dotted lines in Figure 12. The legend “Hex & Central & Tx” represents the simulation results when the central antenna is configured as a calibration signal transmitter for arrays with hexagonal topologies. Other legends can be interpreted in a similar fashion.

**Figure 13:**
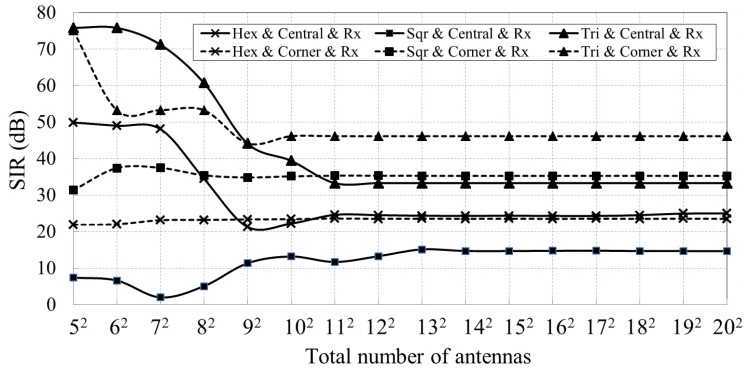
Received calibration signal’s SIR versus total number of antennas, where the solid lines show the calibration signal’s SIR when the central antenna is configured as a receiver. The dotted lines show the calibration signal’s SIR when the antenna at the top left corner is configured as receiver.

Furthermore, we turn the central antenna and interference transmitters into receiving mode. Their one-hop antennas are activated in transmitting mode. This situation corresponds to the transmitter path calibration in Section 2.3. The received calibration signal’s SIR of the central antenna is shown as the solid lines in Figure 13. The received calibration signal’s SIR of the receiver located at the top left corner of the array is shown as the dotted lines in Figure 13.

Figure 12 and Figure 13 reveal that the calibration signal’s SIR stays stable for arrays with at least 12×12 antennas, regardless of the array topology. The lowest SIR is about 14 dB, which is sufficient to perform the REV calibration method. The simulation results also suggest that our proposed method can perform well for arrays with at least 12×12 antennas, while it may not be able to achieve good results when the array is small.

### 4.3. Calibration Time Slots vs. Total Number of Antennas

**Figure 14:**
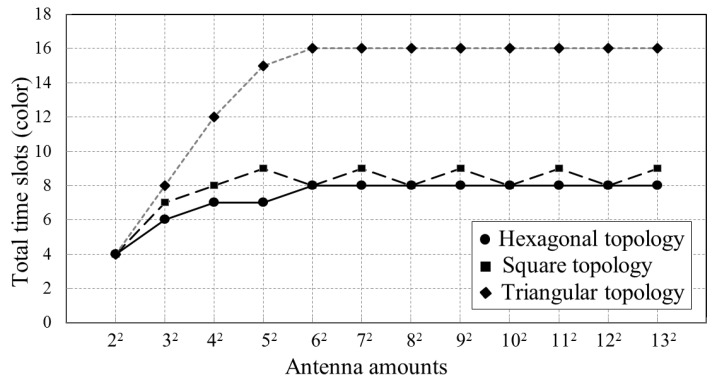
Simulation results of total time slots used for arrays with different number of antennas and different topologies.

In the third simulation, we simulate the required time slots versus the antenna number on arrays with different scales and topologies in Microsoft Visual Studio 2010. The simulation results in Figure 14 show that the total time slots increase with antenna number increasing when array scale is small. However, when the antenna scale continues to grow, the required time slots quickly saturate. The maximal required time slots used by arrays with hexagonal topology, square topology, and triangular topology are only 8, 9, and 16, respectively. As a comparison, we list time slots used by other methods and the proposed method in Table 5. The proposed method requires minimal time slots among all mentioned methods.

**Figure 15:**
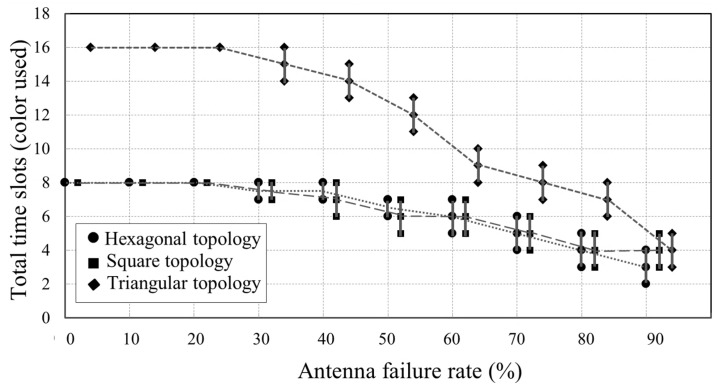
Simulation of the total time slots for arrays with different antenna failure rates.

Figure 15 presents the simulated total calibration time slots used for 12×12 arrays with different antenna failure rates. It can be seen that the total time slots reduce as the antenna failure rate increases. This is because the calibration time slot available for an antenna is confined by its neighbors within three-hops. However, when some of its neighbor antennas fail, the antenna loses mutual-coupling-based connections, which means more calibration time slots belonging to its neighbors become available.

**Figure 16:**
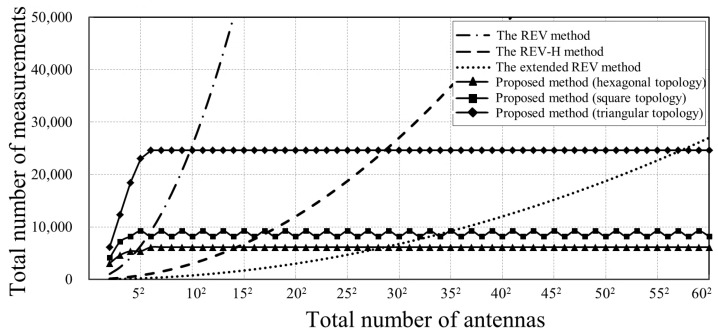
Comparison of the total number of measurements between the REV method, the REV-H method, the extended REV method and the proposed method with different topologies.

Figure 16 compares the total number of measurements consumed by different calibration methods. The REV-H method consumes fewer measurements compared to the REV method, which requires only 30 phase shifting operations per calibration time slot. In addition, its total calibration time slots equal to the number of antennas. Hence, the total number of measurements is 30N. The total number of measurements with the extended REV method is at least 7.5N+1.

Exploiting the graph coloring algorithm, the proposed method reduces the calibration time slots from *N* to no more than 16 for the three topologies mentioned above. Therefore, the total number of measurements is 256KP, where *P* represents total calibration time slots used and is shown in Figure 14. *K* represents the repetitions of phase shift in each calibration time slot, which depends on array topology. Take a large planar array with square topology as an example. When an antenna is activated to perform mutual coupling-based REV calibration, its 4 one-hop neighbors sequentially repeat the 256-steps phase shifting, where *K* = 4. Similarly *K* = 3 and 6 for hexagonal topology and triangular topology respectively. Simulations suggest that the proposed method demonstrates a great advantage when *N* is large, as shown in Figure 16. The total number of measurements required by other methods is also summarized in Table 6.

**Figure 17:**
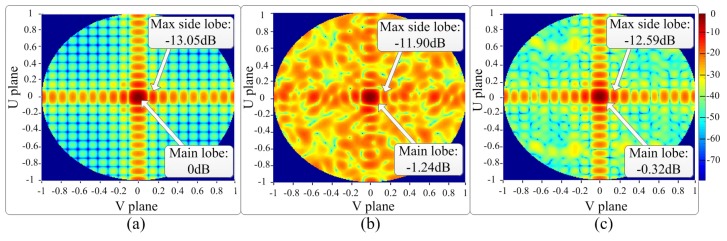
The contour plots of the radiation pattern of a planar array with 12×12 antennas. The main beam locates at the center of every plot. The relative power intensity is indicated by different colors. (**a**) The radiation pattern without any amplitude and phase errors. (**b**) The radiation pattern with antenna amplitude and phase errors. (**c**) The radiation pattern calibrated by the proposed method. SIR = 14 dB.

The radiation pattern comparison before and after our proposed calibration is shown in Figure 17. A 12×12 square-tilted planar array is used in this comparison. Initially, every antenna is identical without any amplitude and phase errors. The radiation pattern of this ideal phased array is shown in Figure 17a. The maximal side lobe power is 13.05 dB lower than the main lobe beam. The plot is central symmetric perfectly. Then every antenna is assigned with random amplitude and phase errors. The standard deviations of amplitude and phase errors are 3 dB and 120 degrees, respectively. The radiation pattern is shown in Figure 17b. Without any calibration, the main lobe power deteriorates by 1.24 dB compared to Figure 17a, and the maximal side lobe power is 1.15 dB higher than the side lobe in Figure 17a. Furthermore, the average power of the side lobes is much higher than Figure 17a (the green and dark blue areas in Figure 17a turn into red and yellow in Figure 17b). To calibrate this phased array, the proposed calibration time allocation method is implemented on this phased array. Hence, each antenna is assigned to a certain time slot. As a result, multiple antennas perform local calibration processes at the same time slot to obtain the amplitude and phase errors among their one-hop neighbors. The SIR of each calibration signal is shown in Figure 12 and Figure 13. After all antennas have been calibrated, the system obtains amplitude and phase errors among all antennas. These known amplitude and phase errors are further trimmed out by electronic circuits or digital signal processing. The radiation pattern after calibration is shown in Figure 17c. The recovered main lobe is 0.92 dB higher than that in Figure 17b, and the maximal side lobe power is improved by about 0.69 dB. Also, the average sidelobe power is much lower than that in Figure 17b.

## 5. Conclusions

We have described a graph coloring theory-based method to calibrate omnidirectional phased array antennas with a high speed. We adopted the graph coloring theory to transform the calibration scheduling problem into a coloring problem. To validate, we performed several simulations to compare our proposed method with several other calibration methods. Simulation results show that our method can prominently reduce total calibration time. Further simulations indicate that the proposed method can recover the radiation pattern from amplitude and phase errors.

## Figures and Tables

**Table 1:** The neighbor map of 9 antennas after the first round broadcasting.

Antenna No.	One-Hop Neighbors	Two-Hops Neighbors	Three-Hops Neighbors
1	2	Null	Null
2	1, 3, 4	Null	Null
3	2	Null	Null
4	2, 5	Null	Null
5	4, 6, 8	Null	Null
6	5, 7	Null	Null
7	6, 9	Null	Null
8	5	Null	Null
9	7	Null	Null

**Table 2:** The neighbor map of 9 antennas after the second round broadcasting.

Antenna No.	One-Hop Neighbors	Two-Hops Neighbors	Three-Hops Neighbors
1	2	3, 4	Null
2	1, 3, 4	5	Null
3	2	1, 4	Null
4	2, 5	1, 3, 6, 8	Null
5	4, 6, 8	2, 7	Null
6	5, 7	4, 8, 9	Null
7	6, 9	5	Null
8	5	4, 6	Null
9	7	6	Null

**Table 3:** The neighbor map of 9 antennas after the third round broadcasting.

Antenna No.	One-Hop Neighbors	Two-Hops Neighbors	Three-Hops Neighbors
1	2	3, 4	5
2	1, 3, 4	5	6, 8
3	2	1, 4	5
4	2, 5	1, 3, 6, 8	7
5	4, 6, 8	2, 7	1, 3, 9
6	5, 7	4, 8, 9	2
7	6, 9	5	4, 8
8	5	4, 6	2, 7
9	7	6	5

**Table 4:** The simulated mutual coupling results at 2.33 GHz with different *S*.

Distance, *S*	Mutual Coupling (S21)
*d*	−17.34 dB
$\sqrt{2}d$	−27.45 dB
$\sqrt{3}d$	−32.35 dB
2d	−35.90 dB
$\sqrt{7}d$	−44.86 dB
3d	−50.56 dB
$\sqrt{13}d$	−57.94 dB
4d	−63.50 dB
5d	−76.83 dB
6d	−90.05 dB

**Table 5:** Comparison of time slots used among different methods, where *N* is the total number of antennas.

Method	Total Time Slots
The REV method	*N*
The extended REV method	*N*
The REV-H method	*N*
Proposed method(hexagonal topology)	≤8 *
Proposed method(square topology)	≤9 *
Proposed method(triangular topology)	≤16 *

* Refer to Figure 14 for specific total time slots used by arrays with various *N*.

**Table 6:** Comparison of total number of measurements of a large array with *N* antennas by different methods.

Method	Total Number of Measurements
The REV method	256N
The REV-H method	30N
The extended REV method	7.5N+1
Proposed method(hexagonal topology)	≤6144 *
Proposed method(square topology)	≤9216 *
Proposed method(triangular topology)	≤24,576 *

* Refer to Figure 16 for specific total number of measurements used by arrays with various *N*. Besides, it can be calculated by the expression mentioned above, 256KP.
